# Supplementary material for: Codon deoptimization of multispecific biologics reduces mispairing during transient mammalian protein expression
Source: Front Bioeng Biotechnol. 2026 Feb 26;14:1783067. doi: 10.3389/fbioe.2026.1783067 (PMC12980028; doi:10.3389/fbioe.2026.1783067)
Supplement: Supplementary file 1 [file Image2.pdf]

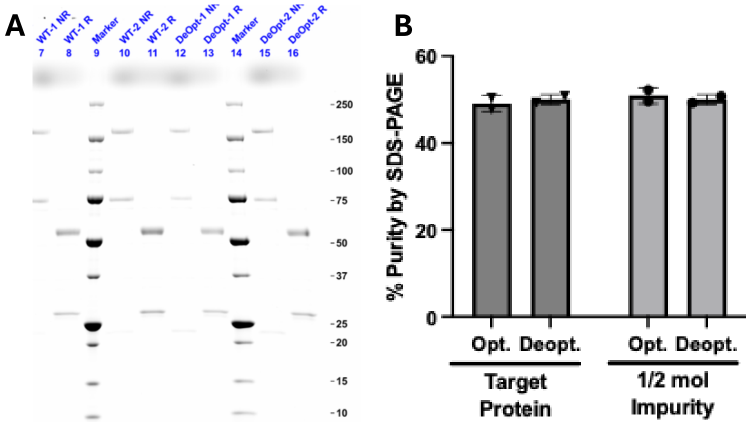

Figure S2. (A) Non-reduced and reduced SDS-PAGE of conditioned media (CM) from optimized (WT) and deoptimized expressions of Protein 2. (B) SDS-PAGE band quantitation of the target protein and half molecule bands in optimized and deoptimized expressions.
