# Supplementary material for: Codon deoptimization of multispecific biologics reduces mispairing during transient mammalian protein expression
Source: Front Bioeng Biotechnol. 2026 Feb 26;14:1783067. doi: 10.3389/fbioe.2026.1783067 (PMC12980028; doi:10.3389/fbioe.2026.1783067)
Supplement: Supplementary file 3 [file Image1.pdf]

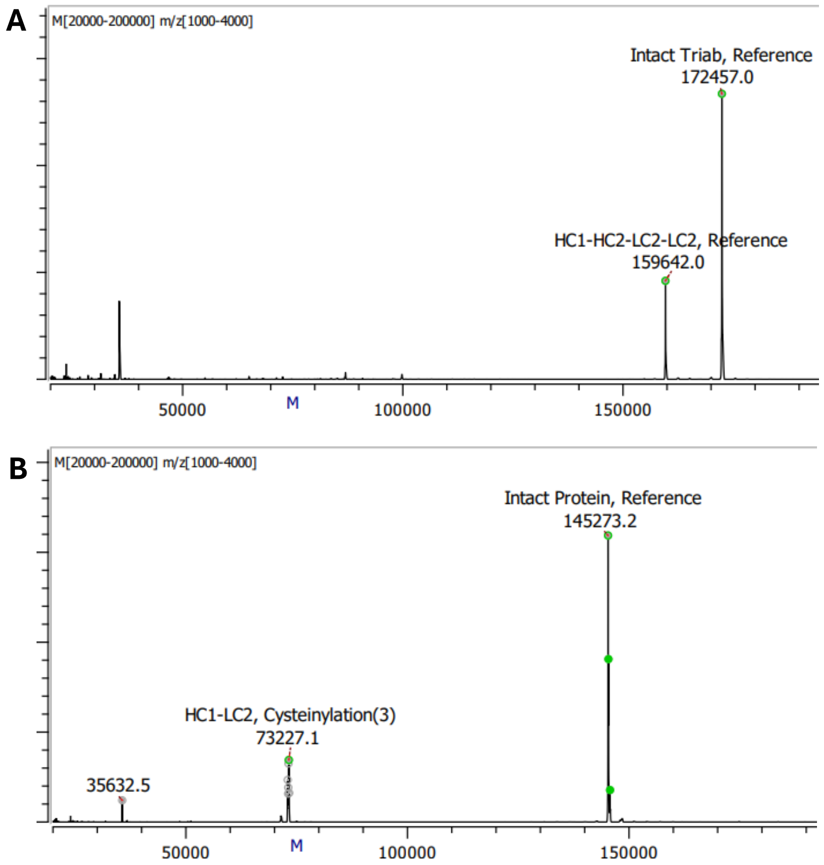

Figure S1. Representative mass spectra of the CODV trispecific Protein 1 (A) and the bispecific protein (B) with their associated impurity peaks.
